# Supplementary material for: Progression of the late-stage divisome is unaffected by the depletion of the cytoplasmic FtsZ pool
Source: Commun Biol. 2021 Mar 1;4:270. doi: 10.1038/s42003-021-01789-9 (PMC7921118; doi:10.1038/s42003-021-01789-9)
Supplement: Supplementary file 2 — Description of Additional Supplementary Files [file 42003_2021_1789_MOESM2_ESM.pdf]

Description of Additional Supplementary Files

File name: Supplementary Movie 1

Description: Time-lapse video of exponentially growing B. subtilis 2020 cells treated with 0.25 µg/ml ADEP2 (compare Fig. 2a). Overlaid fluorescence and phase contrast images show the localization of GFP-tagged FtsZ (in green). Early FtsZ rings disintegrate during ADEP treatment (open triangles) while more progressed FtsZ rings constrict and finish septum formation (closed triangles).

File name: Supplementary Movie 2

Description: Time-lapse video of exponentially growing B. subtilis 2020 cells treated with 0.25 µg/ml ADEP2 (compare Supplementary Fig. 2). Overlaid fluorescence and phase contrast images show the localization of GFP-tagged FtsZ (in green). Early FtsZ rings disintegrate during ADEP treatment (open triangles) while more progressed FtsZ rings constrict and finish septum formation (closed triangles).

File name: Supplementary Movie 3

Description: Time-lapse video of exponentially growing B. subtilis 2020 cells treated with 0.25 µg/ml ADEP2 (compare Supplementary Fig. 2). Overlaid fluorescence and phase contrast images show the localization of GFP-tagged FtsZ (in green). Early FtsZ rings disintegrate during ADEP treatment (open triangles) while more progressed FtsZ rings constrict and finish septum formation (closed triangles).

File name: Supplementary Movie 4

Description: Time-lapse video of exponentially growing B. subtilis CM03 cells treated with 0.125 µg/ml ADEP2 (compare Fig. 3a). Overlaid fluorescence and phase contrast images show the localization of mCherry-FtsZ (in red) or GFP-PBP2b (in green), as indicated, during ADEP treatment over time. Early divisomes disintegrate during ADEP treatment (open triangles) while more progressed divisomes constrict and finish septum formation once PBP2b has formed clear foci (closed triangles).
